# An Exploratory Study of Kinase Activation Profiles in Hypoxic Human Cardiomyocytes Treated with Protective Extracellular Vesicles

**DOI:** 10.21203/rs.3.rs-8557214/v1

**Published:** 2026-01-21

**Authors:** Andreas Czosseck, Barbara Szomolay, Ali Sajid Imani, Robert E. McCullumsmith, Patrick C.H. Hsieh, Thierry Burnouf, David J. Lundy

**Affiliations:** Taipei Medical University; Cardiff University School of Medicine; University of Toledo; University of Toledo; Academia Sinica; Taipei Medical University; Taipei Medical University

**Keywords:** Exosome, kinome, pamstation, phosphorylation, induced pluripotent stem cells

## Abstract

**Purpose:**

Myocardial infarction (MI) causes hypoxic cardiomyocyte death, and extracellular vesicles (EVs) offer therapeutic potential. This study aimed to compare kinase activation profiles induced by four human-derived EV types-serum-derived (S-EVs), platelet concentrate-derived (PC-EVs), cardiac stromal cell-derived (CSC-EVs), and bone marrow mesenchymal stromal cell-derived (MSC-EVs)- in hypoxic metabolically matured human iPSC-derived cardiomyocytes (iCMs).

**Methods:**

Metabolically matured human induced pluripotent stem cell-derived cardiomyocytes (iCMs) were exposed to 12-hour hypoxia ± standardized doses of EVs. Kinase activity was profiled using the PamStation platform, and bioinformatic tools (KRSA, UKA, PTM-SEA, KEA3) identified differentially activated kinases. AKT phosphorylation (Ser473) was measured by ELISA.

**Results:**

All EVs significantly reduced LDH release versus hypoxia alone (P ≤ 0.0001), with no inter-group differences. Hypoxia globally suppressed kinase activity, while each EV type induced distinct patterns: S-EVs and PC-EVs increased total phosphorylation, whereas CSC-EVs and MSC-EVs further decreased it. Bioinformatics implicated the AKT family in all treatments, but ELISA revealed no change in AKT1/2/3 phosphorylation at Ser473 versus hypoxia controls.

**Conclusion:**

Despite equivalent cardioprotection, each EV type elicited unique kinase activation profiles, suggesting distinct signaling mechanisms. Kinase activity was not a predictor of protection at the measured time point, highlighting the complexity of EV-mediated pathways.

## Background

Myocardial infarction (MI) is a leading cause of mortality worldwide, resulting from hypoxic injury of cardiac tissue. Cardiomyocytes (CMs) are highly dependent on oxygen for ATP generation via fatty acid oxidation, thus hypoxia results in rapid CM death, leading to reduced cardiac contractile function. Preservation of viable CMs during and after MI is an important therapeutic goal, since these cells cannot proliferate in meaningful amounts to regenerate injured myocardium. Small molecules, peptides, live cells, and components of the cell secretome (growth factors, cytokines *etc*.) have been previously explored for their potential to reduce or prevent cardiomyocyte death during or following hypoxic conditions ^1^.

Extracellular vesicles are lipid membrane bound vesicles secreted by cells and have become emerging candidates for acute cardioprotection. Studies in cell and animal models have shown that EVs can offer equal or superior efficacy as transplanting live therapeutic cells, with less limitations relating to donor cell survival or safety ^2,3^. Cardioprotection by EVs has been demonstrated across multiple *in vitro* and *in vivo* models of ischemia/reperfusion injury in multiple species, using EVs from a wide variety of sources ^4–7^ .

In this exploratory study we utilize EVs isolated from four clinically-relevant human sources; serum (S-EVs), platelet concentrates (PC-EVs), cultured cardiac stromal cells (CSC-EVs), and cultured bone marrow mesenchymal stromal cells (MSC-EVs). These four EV types were selected because they have all previously shown CM-protective effects and have well characterized proteomic and miRNA cargo profiles ^8–12^. Some prior studies have conducted direct, head-to-head comparisons of EVs from different sources in standardized models of cardiomyocyte hypoxia, showing that EV protective abilities can vary between sources ^9,13,14^. This is likely because EVs contain complex cargos comprising thousands of proteins, and hundreds of unique miRNAs per EV population ^9–11,15^. Proposed mechanisms of EV cardioprotective actions are heterogenous, including nucleic acid (mRNA, miRNA *etc.*) or peptide delivery, EV-cell surface protein interactions, delivery of metabolites, transfer of mitochondria, or extracellular chelating functions ^6,16–24^. Proteomic and transcriptomic studies of EV-treated CMs have identified hundreds of differentially expressed genes and proteins. This demonstrates that EVs are capable of modulating several intracellular pathways, including those responsible for responses to oxidative stress and anti-apoptosis ^9,25^.

Kinase signaling pathways are central to CM proliferation, survival and stress responses, particularly during hypoxia ^26–28^. Several key pathways, including the MAPK, ERK, and AKT pathways, have been identified as critical mediators of cardiomyocyte injury or recovery following hypoxic stress ^29–31^. In particular, the PI3K/AKT pathway is a well-known regulator of CM survival and metabolism, and increased AKT phosphorylation has been demonstrated in response to cardioprotective therapies ^32^. For example, a recent study showed, using proteomics, that iPSC-derived EVs protected CMs from hypoxia by enhancing the AKT/ERK/NRF2 pathway ^25^. The cardiac kinome has been previously explored using transcriptomics ^33^. However, effects of EVs on CM kinase enzyme activity remain unexplored. To address this, in the current study, we opted for an alternative approach, using Pamgene kinome technology to simultaneously profile hundreds of kinases in an unbiased manner. Importantly, the assay measures kinase activity in realtime rather than gene expression levels, protein concentrations or phosphorylation states as a proxy for protein kinase activity. Thus, the array provides an effective snapshot of the signaling networks in the cells.

Here, our strategy was to compare the effects of four therapeutic human-derived EV types: S-EVs, PC-EVs, CSC-EVs, and MSC-EVs, in a well controlled *in vitro* model of MI, using human iPSC-derived cardiomyocytes (iCMs) ^34^. To make our study more physiologically relevant, the iCMs were first metabolically matured to more closely reflect adult human CMs, and enhance their sensitivity to hypoxia ^35–37^. We then applied unbiased, open-source bioinformatics approaches to compare the experimental groups. Our primary aims were to determine whether these EVs influenced CM kinase activity during hypoxia, and to assess whether EVs mediate their effects through common or distinct signaling pathways. In comparing the EVs isolated from different sources, we elected to use the optimal isolation methods for each source, and to compare EV activities at a fixed single dose, which was normalised to both protein and particle concentrations.

## Results

### Isolating and characterizing human extracellular vesicles from four sources

**Figure 1:**
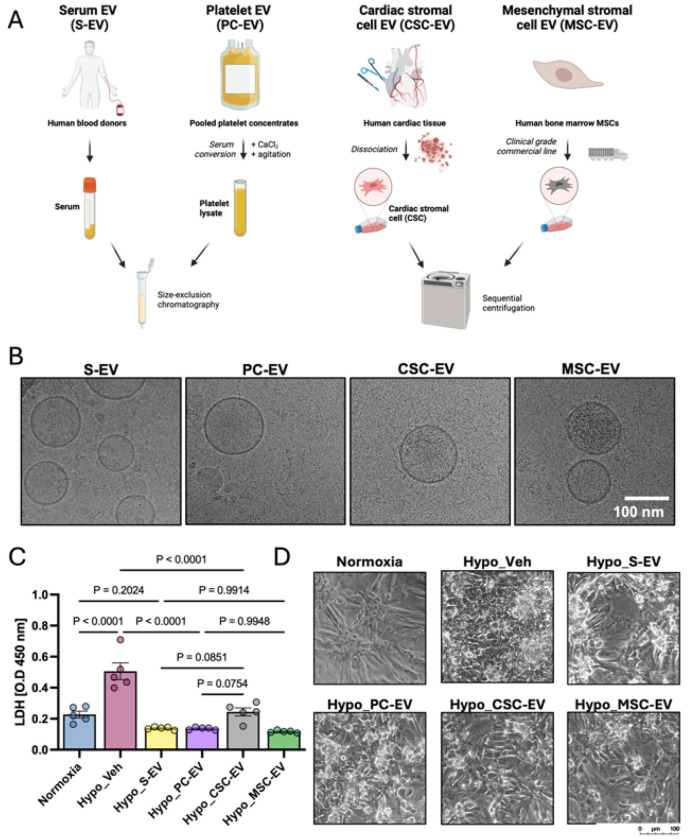
EV isolation and assessment of cardioprotection (A) Schematic diagram showing processes of EV isolation from four human source materials. (B) Representative CryoEM images of EVs from each source. (C) Quantification of lactate dehydrogenase (LDH) release from metabolically matured iCMs. Cells were cultured in normoxia, hypoxia + vehicle control (Hypo_veh), or hypoxia with S-EVs, PC-EVs, MSC-EVs or CSC-EVs. Data are shown as OD at 450 nm, with blank values subtracted. Each individual data point is shown, and error bars show standard error of the mean. Samples were compared by one-way ANOVA with Tukey’s post-test, with P values as indicated. (D) Representative light microscopy images of iCMs from each condition. Scale bar 100 μm.

A schematic diagram of the EV isolation process is shown in Figure 1A and properties of the EV isolates are shown in Table 1. Each EV type was isolated using best practice methods for each original sample type, with UC used for the large volumes of conditioned culture medium, and SEC used for the smaller volumes of concentrated serum and platelet concentrates. The end goal was to produce representative therapeutic EV preparations from each source. Nanoparticle tracking analysis (NTA) showed that populations of nanoscale particles were successfully isolated from all samples, with mean diameters ranging between 112.9 nm (PC-EVs) and 162.1 nm (MSC-EVs), which are within typical 100 – 200 nm diameters expected of EV preparations ^38^. In terms of EV yields, PCs and serum contained significantly higher particle concentrations and protein content per ml of original sample than culture-derived EVs, as expected ^39,40^. In particular, clinical PCs are 4-5x more concentrated than physiological levels, thus producing higher yields. There was no significant difference between the yields of EVs from cultured MSCs or CSCs, consistent with our previous findings ^9^. The purity of all isolated EVs exceeded 1x10^10^ particles per μg of total EV protein, indicating high purity ^38^. Lastly, cryoEM was used to visualize each sample, showing lipid bilayer membrane-bound spherical vesicles, thus confirming the presence of EVs in each preparation (Figure 1B). In previous studies, using the same source materials and isolation methods, we have shown that the samples contain CD81, CD9, Alix, TSG-101 and other EV markers ^10,11,41^. Thus, taken together, these data confirm EVs were successfully isolated.

### Confirmation of induced pluripotent stem cell-derived cardiomyocyte maturation

iPSC-derived cardiomyocytes (iCMs) were used as the platform for hypoxic injury modeling. Flow cytometry for cardiac troponin I showed >90% positivity (**Supplemental Figure 1A**), indicating a high purity of iCMs. Following culture in FA-medium the MYH6/MYH7 ratio significantly increased (**Supplemental Figure 1B**) ^42^. An additional panel of genes (**Supplemental Figure 1C**) showed significant downregulation of glycolytic and fatty acid synthetic enzymes, downregulation of fetal CM marker NPPA, and increased expression of fatty acid oxidation enzymes. Together, these data demonstrate that iCMs were successfully matured ^36,42,43^. Next, iCMs were exposed to hypoxia and nutrient deprivation injury to simulate MI. A time course of cell survival after different timepoints is shown in **Supplemental Figure 2A**, showing that the matured cells were highly sensitive to hypoxia ^35^. Based on these results, 12h hypoxia (**Supplemental Figure 2B**) was selected for the following experiments, since we aimed to induce severe iCM injury without excessive cell death.

### Assessing ability of extracellular vesicles to protect hypoxic iCMs

iCMs were incubated with equal doses of either S-EVs, PC-EVs, CSC-EVs or MSC-EVs, or with an equivalent volume of PBS (vehicle) as a control. The EV concentration was selected based on previous studies showing efficacy around this dose range.^9–11^ Parallel cultures were maintained in normoxia in the same basal medium to serve as healthy (negative) controls. LDH was used as a sensitive metric of cumulative iCM injury, as previously described ^37^. Quantification of LDH release is shown in Figure 1C. LDH release significantly (2.3-fold, P ≤ 0.0001) increased with hypoxia + vehicle (hypo_veh) compared to normoxia, as expected ^37^. All of the EV treatments significantly (P ≤ 0.0001) reduced LDH release compared to the vehicle control, and there were no significant differences between the four EV treatments. We noted that CSC-EV-treated iCMs had higher, more variable, LDH release, but this was not significantly different to other groups, and LDH release was still significantly lower (P ≤ 0.0001) than hypoxia + vehicle. These results were supported by cell morphology (Figure 1D), which was notably disrupted by hypoxia, showing shrunken, spherical cells, detachment from the culture surface, and degraded connections between clusters of iCMs. On the other hand, all EV treatments showed greater preservation of typical iCM morphology. Lastly, the total protein content of cell lysate was measured (**Supplemental Figure 2C**). Here, small differences were observed where hypoxia increased total protein levels, while some of the EV groups reduced this to the level of normoxic cells. Together, the results showed that the four EV sources were equally protective of metabolically matured iCMs during hypoxia.

### Profiling levels of kinase enzyme activity in normoxic and untreated hypoxic iCMs

In total, 24 samples were processed across two separate runs, as shown in **Supplemental Figure 3A**. Samples were laid out so that each chip contained negative (normoxic) and positive (hypoxia + vehicle) control samples, and all samples passed QC checks based on sufficient signal intensity and linearity. All samples had low coefficients of variation (CV%) (**Supplemental Figure 3B**) with consistent signal intensities across the replicates (**Supplemental Figure 3C**).

**Figure 2:**
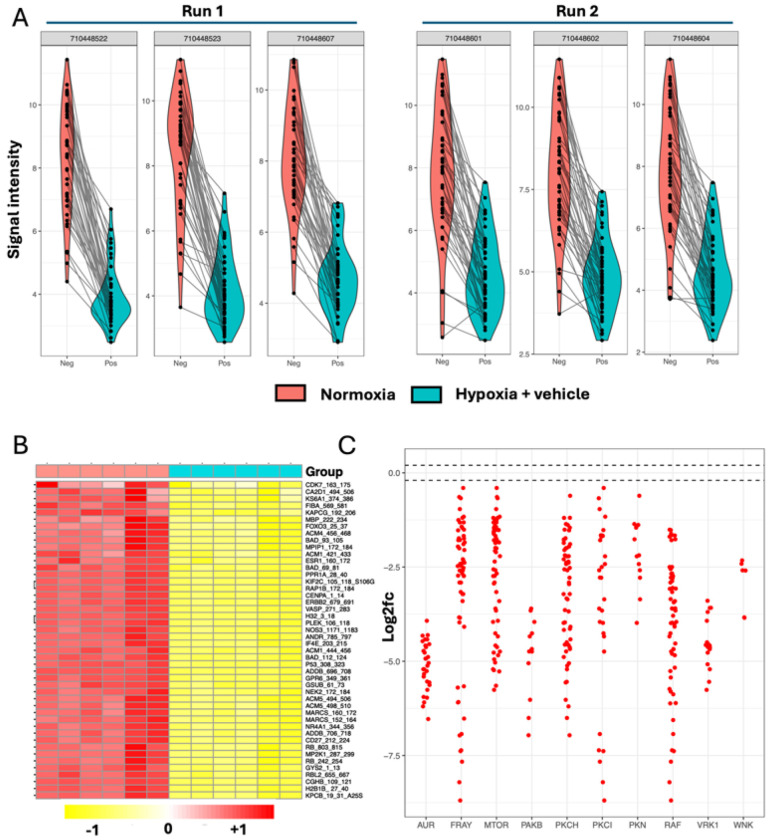
Hypoxia induces global reductions in kinase activity in untreated iCMs (A) Violin Plots showing signal intensity distribution (Y axis) for the six replicates of normoxia (neg) vs. hypoxia + vehicle (pos) treated iCMs. Left; run 1, Right; run 2. The grey numbers represent the unique barcode for each chip and each data point shows the signal intensity of peptide phosphorylation. (B) Heatmap showing normalised peptide phosphorylation levels in normoxia (n = 6) vs. hypoxia + vehicle (n = 6) iCM samples. 46/141 peptides passed filtering parameters. Unsupervised hierarchical clustering was applied as shown on the X and Y axes. Peptide names are annotated to the right of the heatmap. (C) Reverse KRSA plot of kinase families showing mean of six samples. The Y axis shows log2fc of hypoxia + vehicle vs. normoxic iCMs with log2fc = 0 indicating no change. Dotted lines at +0.2 and −0.2 indicate thresholds for significance.

Comparing normoxic iCMs (neg) against hypoxia + vehicle iCMs (pos) at the peptide phosphorylation level, hypoxia produced a strong reduction in phosphorylation of all peptides, as shown by violin plots (Figure 2A). Since neg vs. pos comparisons were performed in two separate runs, these were pooled for a six vs. six analysis. The heatmap of normalized intensity values (Figure 2B) shows 46 peptides which crossed signal thresholds, showing strong downregulation of peptide phosphorylation in the hypoxia + vehicle-treated iCMs. Unsupervised hierarchical clustering of 12 total samples separated the results into the two experimental conditions, indicating a large biological difference between the two groups. The differential phosphorylation levels of each peptide were then mapped to their upstream kinases using ‘reverse KRSA’, which were grouped into families, with activity shown as log2fc compared to normoxia (i.e. log2fc of 0), as in Figure 2C. With hypoxia + vehicle control, all kinase activities were decreased compared to normoxia, far exceeding ± 15% changes, which is the conventionally accepted threshold for biological significance in this array ^44^. KRSA upstream kinase analysis scores are shown in Table 2. By mapping kinases which can phosphorylate each peptide on the chip, a list is generated and compared to a random sampling approach ^45^.

### Profiling levels of iCM kinase enzyme activity during hypoxia with EVs

**Figure 3:**
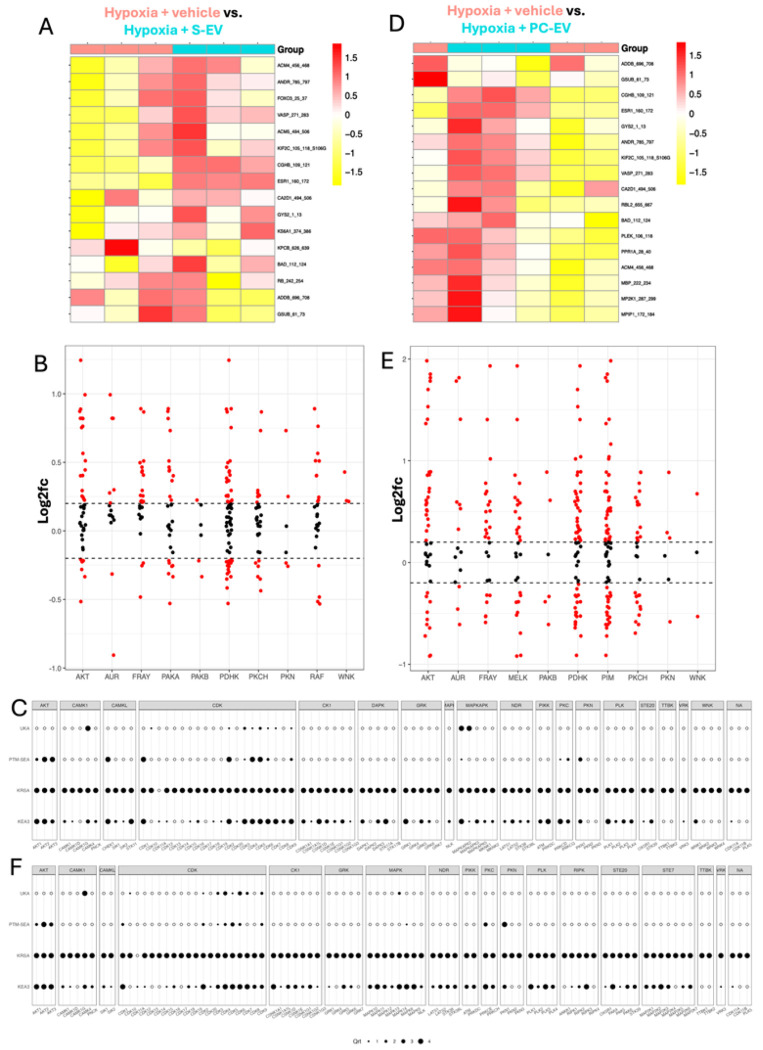
Effects of S-EVs and PC-EVs on kinase activity in hypoxic iCMs (A) Heatmap showing normalized peptide phosphorylation levels in hypoxia + vehicle vs. hypoxia + S-EV-treated iCMs. 16/141 peptides passed filtering parameters. Unsupervised hierarchical clustering was applied as indicated. (B) Reverse KRSA kinase activity summary for hypoxia + vehicle vs. hypoxia + S-EV-treated iCMs, grouped by kinase families. The Y axis shows log2fc with log2fc = 0 indicating no change. Dotted lines at +0.2 and −0.2 indicate thresholds for significance. Points above and below the thresholds are indicated in red. (C) Creedenzymatic plot for hypoxia + vehicle vs hypoxia + S-EV-treated iCMs. Kinase families are shown in grey boxes on the upper X axis, and individual enzymes are shown underneath. UKA, PTM-SEA, KRSA and KEA3 were used to assess significance, as labelled on the left. For each combination, a percentage rank normalization is used and assigned a value 1-4, with 4 being the most significant. The legend is shown below the plots. (D) Heatmap for hypoxia + vehicle vs. PC-EV-treated iCMs. 17/141 peptides passed filtering parameters. (E) Kinase activity summary for hypoxia + vehicle vs. hypoxia + PC-EV-treated iCMs. (F) Creedenzymatic plot for hypoxia + vehicle vs. hypoxia + PC-EV-treated iCMs.

**Figure 4:**
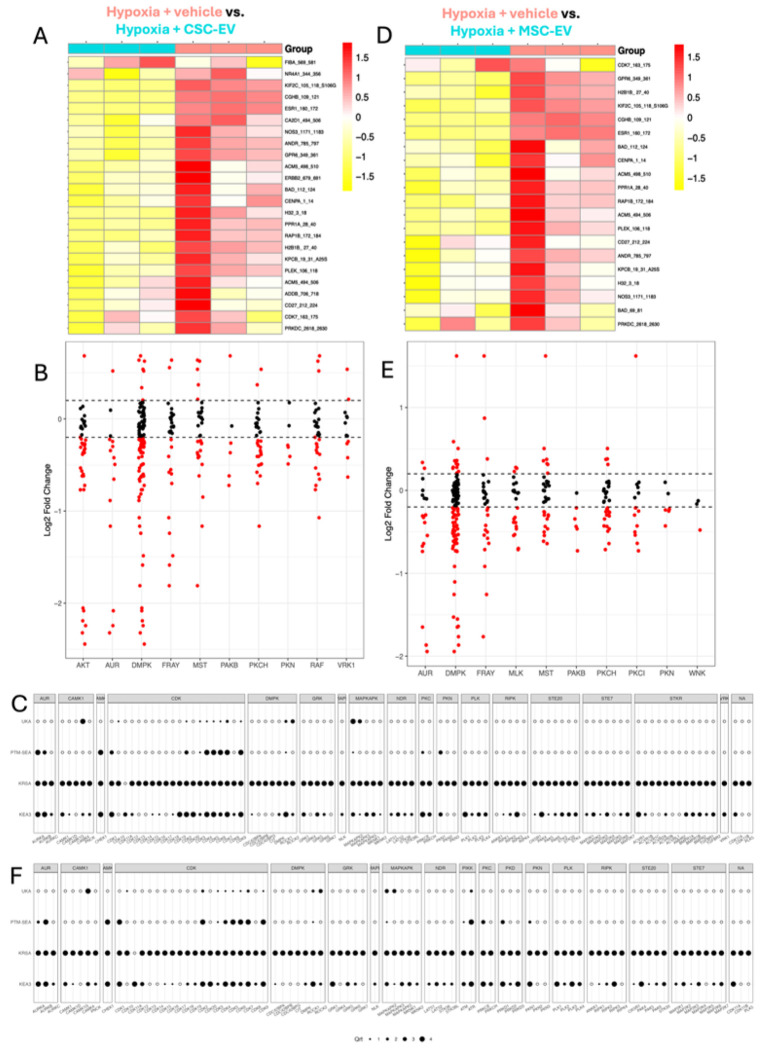
Effects of CSC-EVs and MSC-EVs on kinase activity in hypoxic iCMs (A) Heatmap showing normalized peptide phosphorylation levels in hypoxia + vehicle vs. hypoxia + CSC-EV-treated iCMs. 24/141 peptides passed filtering parameters. Unsupervised hierarchical clustering was applied as indicated. (B) Reverse KRSA kinase activity summary for hypoxia + vehicle vs. hypoxia + CSC-EV-treated iCMs, grouped by kinase families. The Y axis shows log2fc with log2fc = 0 indicating no change. Dotted lines at +0.2 and −0.2 indicate thresholds for significance. Points above and below the thresholds are indicated in red. (C) Creedenzymatic plot for hypoxia + vehicle vs. hypoxia + CSC-EV-treated iCMs. (D) Heatmap for hypoxia + vehicle vs. hypoxia + MSC-EV-treated iCMs. 20/141 peptides passed filtering parameters (E) Kinase activity summary for hypoxia + vehicle vs. hypoxia + MSC-EV-treated iCMs (F) Creedenzymatic plot for hypoxia + vehicle vs. hypoxia + MSC-EV-treated iCMs

Next, we compared hypoxia + vehicle (pos) treated iCMs against each of the four EV treatment groups; S-EV and PLT-EV-treated iCMs are shown in Figure 3 and CSC-EV and MSC-EV-treated iCMs are shown in Figure 4. The EV samples were compared to the Pos samples in the same run to reduce the impact of between-run variability on the comparison. Since all of the EVs had successfully protected the iCMs (Figure 1C,D), we anticipated that they would normalize or preserve kinase activity, thus showing a profile more similar to normoxic iCMs. However, the results instead showed that each EV treatment induced distinct effects on different kinase families. Comparing total phosphorylation levels (**Supplemental Figure 4**) showed that lysates from iCMs treated with S-EVs and PC-EVs generally increased peptide phosphorylation, while those from MSC-EV and CSC-EV-treated iCMs further decreased phosphorylation compared to vehicle-treated iCMs.

Comparing peptide phosphorylation in hypoxia + vehicle vs. S-EV-treated iCMs (i.e. T1 vs. pos) showed clustering into two groups matching the experimental conditions (Figure 3A), with a mixture of increased and decreased phosphorylation levels. Based on the reverse KRSA plot (Figure 3B), AKT and PDHK kinase activity were strongly increased by S-EV treatment. The creedenzymatic tool was then used to integrate determinations of significance from four bioinformatic tools - UKA, PTM-SEA, KRSA, and KEA3 - as indicated on the left Y axis ^45–47^. For each kinase (shown on bottom X axis), a percentile ranking is given from 1-4, with 4 being the highest significance. Thus, enzymes which show a high ranking across multiple tools are most likely to be biologically important differences between the two treatment groups. Based on the plots (Figure 3C), AKT1-3, CDK4, CDK5, CDK9, MAPKAPK2 were ranked as significant in S-EV activity based on all four prediction tools.

Comparing hypoxia + PC-EV-treated iCMs vs. hypoxia + vehicle (T2 vs. pos) treated iCMs did not show complete hierarchical clustering into experimental groups (Figure 3D), likely due to the third replicate of the PC-EV group which showed lower average phosphorylation levels (**Supplemental Figure 4**). Nevertheless, PC-EVs overall significantly increased phosphorylation of peptides associated with AKT and PDHK families (Figure 3E), similar to S-EVs, but with additional increased PIM activity. Creedenzymatic analysis (Figure 3F) showed similar results to S-EVs, including significant rankings for AKT1-3 and CDKs, with the addition of PKN1. KRSA upstream kinase predictions (Table 2) identified AKT, PKN, FRAY and PAKB as significant for both PC-EVs and S-EVs. Since serum formation involves blood coagulation and releases platelet cargo, similarity between S-EVs and PLT-EVs should be expected.

CSC-EV-treated iCMs and hypoxia + vehicle-treated iCMs showed hierarchical clustering into their experimental groups (Figure 4A), with EV-treated iCMs showing overall decreased peptide phosphorylation (**Supplemental Figure 4**). Reverse KRSA plot (Figure 4B) showed that CSC-EVs decreased AKT activity, which was the opposite to S-EVs and PC-EVs. This is also shown in Table 2, where S-EV and PC-EVs both strongly implicated AKT but CSC-EVs did not. Interestingly, CSC-EVs also induced a strong decrease in DMPK activity compared to hypoxia + vehicle alone. Creedenzymatic plot (Figure 4C) ranked MAPKAPK2 and ROCK1 followed by CDK4, 5, 6, 9 and AURB as significant. This shows a highly distinct profile of kinase activity compared to S-EV or PC-EV treatment.

Hypoxic iCMs treated with MSC-EVs again showed clear hierarchical clustering (Figure 4D) and decreases in overall kinase activity (**Supplemental Figure 4**) compared to hypoxia + vehicle-treated iCMs. Similar to CSC-EV treatment, MSC-EVs induced a decrease in DMPK activity (Figure 4E, F) and MAPKAPK2, ROCK1, and multiple CDKs. Upstream kinase analysis (Table 2) also showed similarity between CSC-EVs and MSC-EVs by implicating PAKB, PKCH and AUR in both treatments. Overall, MSC-EVs had the least effect on hypoxic iCM kinase activity out of the four EV types investigated.

### Assessing similarities and differences in activity of four EV treatments on hypoxic iCMs

**Figure 5:**
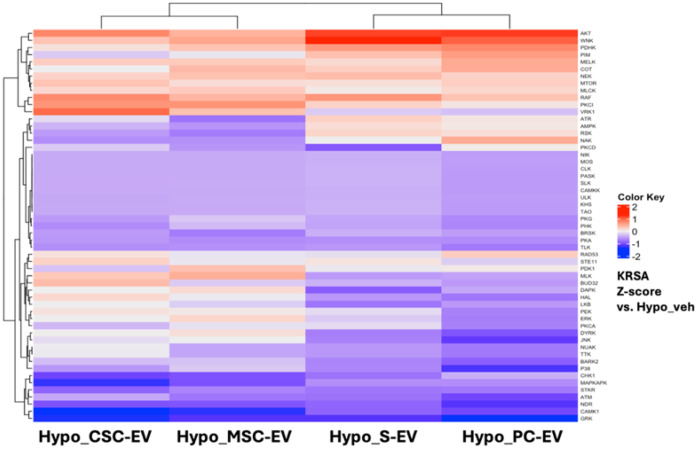
Heatmap comparing four EV treatments against untreated hypoxia iCMs Clustered heatmap comparing four EV treatments against untreated hypoxia iCMs. The colour bar represents the KRSA Z vs hypoxia +vehicle control-treated iCMs. Unsupervised hierarchical clustering was applied, shown on both the upper X axis and Y axis.

Lastly, we compared the effects of the four EV treatments to determine whether they modulated common or differential pathways of activity. A summary heatmap showing KRSA Z-scores compared to control hypoxia + vehicle-treated iCMs is shown in Figure 5. Unsupervised hierarchical clustering showed greater similarity between treatment with cell-derived EVs (MSC-EVs and CSC-EVs) or blood-derived EVs (S-EVs and PC-EVs), however the majority of kinase families were similarly implicated in all four treatments. AKT, WNK and PDHK showed highly positive Z-scores across all treatments. In terms of differences between groups, a cluster of kinases including DAPK, ERK and PKCA, as well as PIM, AMPK, ATR and RSK families had negative Z scores for CSC-EV and MSC-EV-treated iCMs but positive scores for S-EV and PC-EV-treated iCMs. In terms of overall modulation, the AKT family was the strongest implicated by all four treatments.

### Direct measurement of AKT phosphorylation levels

**Figure 6:**
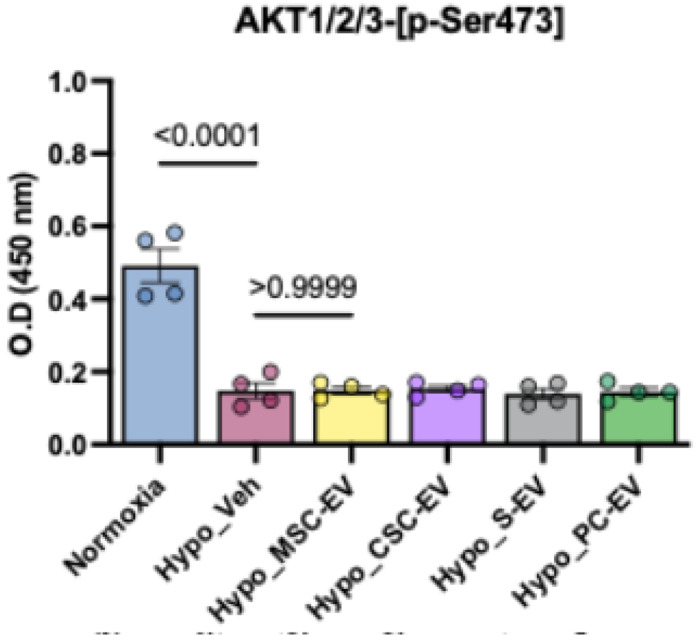
ELISA of AKT1/2/3-p-Ser473 levels Graph showing absorbance at 450 nm from an ELISA. Four independent biological samples per group were tested and results were compared by one-way ANOVA with Tukey’s post-test. P values are indicated on the graph. All treatment groups were P > 0.9999 compared to Hypo_Veh.

To further investigate a role of AKT in the response to EVs, we used ELISA to measure Ser473 phosphorylation levels of AKT1/2/3 in cell lysates from the six treatment groups. The results (Figure 6) showed a significant decrease in AKT phosphorylation during untreated hypoxia, in agreement with the kinome assay. However, the EV-treated groups did not show any significant differences in AKT-p-Ser473 levels compared to hypoxia + vehicle.

## Discussion

This exploratory study confirmed that EVs from human serum, platelet concentrates, MSCs and CSCs can all protect metabolically mature human cardiomyocytes from acute hypoxic injury. These results agree with previous findings from multiple research groups, including our own ^9,10,12,17,19^. Examination of cardiomyocyte kinase activity found that 12h hypoxia caused a significant reduction in total activity, which was significantly altered by EV treatments. Counter to our expectations, we did not find normalization of cardiomyocyte kinase activity by EVs; rather, each EV type induced distinct patterns of activation at the time of measurement. S-EVs and PC-EVs EVs increased total kinase activity, while bone marrow MSC-EVs and CSC-EVs further decreased global activity, although they did display higher activity of some kinase families. Overall, PC-EVs showed the strongest effects by upregulating kinase activity, and MSC-EVs showed the least effects. These results, obtained using an unbiased functional activity assay, again highlight the diversity of EV activities on cells which has previously been shown by proteomic and transcriptomic studies ^9,25^.

In terms of kinase activity, the most repeatedly implicated enzyme families included AKT, CDKs, PAKB, PDHK and PCKH. AKT was ranked as important in the activity of all EV treatments by bioinformatic analysis, but measuring AKT phosphorylation (Ser473) found no differences between the EV treated iCMs and untreated iCMs. This is not necesarrily a contradiction, since the phosphorylation state is determined by both kinase and phosphatase activity. Thus, while AKT signalling is widely demonstrated to be strongly cardioprotective, it may not be necessary for EV-derived hypoxia protection, based on our results ^25,28^. CDKs were also strongly affected by hypoxic injury and by EV treatments and are known to be implicated in hypoxia-driven apoptosis of cardiomyocytes ^48^. Similarly, inhibition of CDKs by small molecules has been shown to improve hypoxic tolerance of myoblasts ^49^. The FRAY subfamily, comprising oxidative stress responsive kinase 1 (OXSR1, or OSR1) and SPS1-related proline/alanine-rich kinase (SPAK) was strongly influenced by PC-EVs and S-EVs. These kinases are downstream of WNK, which were also modulated by EVs (Table 2). These enzymes increase sodium transporters, regulating ion flux and cell swelling ^50^.

It is notable that, despite distinct kinase activation patterns induced by each EV type, all of the EVs offered the same degree of protection to hypoxic iCMs, based on LDH release. For example, MSC-EVs showed the least apparent effects on kinase enzyme activities, yet they still provided excellent protection of hypoxic iCMs. Thus, the results indicate that ser/thr kinase activity is not a strong predictor of effective hypoxia protection, which was unexpected. Another interesting finding is the similarity in iCM kinase activity following treatment with S-EVs and PC-EVs (blood derived) or CSC-EVs and MSC-EVs (cell culture derived). This may reflect different protein/miRNA cargo, or the surface corona/co-isolates of the EVs. Based on previously-published data, PC-EVs and S-EVs are more complex since they originate from multiple cell types present in the blood circulation, whereas MSC/CSC-EVs originate from cell monocultures in less complex cell culture medium ^9–11,51,52^.

Advantages of this study include the use of human iCMs which were metabolically matured, producing more relevant hypoxia responses than common myocyte/myoblast lines such as H9C2, C2C12 or AC16. Additionally, EVs were sourced from therapeutically relevant human sources, and were isolated using reproducible techniques which produced high purity preparations. Rather than use the same method for each source material, we used the most relevant method to produce representative EV preparations. Lastly, the kinase assay was validated and analysed without bias using four separate bioinformatic tools (KRSA, UKA, PTM-SEA, KEA3), with the raw data, scripts and analysis pipelines available for other researchers through Github. At the same time, this exploratory study has several limitations. The first is that we assessed the therapeutic effects of EVs based on iCM damage, survival and morphology. In these respects, all EV treatments were equally effective at preserving iCM viability during hypoxia. The lack of cumulative LDH release gives high confidence that the EVs indeed protected the cells for the entire duration of hypoxia. However, since each EV type activates different intracellular pathways, it is possible that other aspects of CM performance (contractility, electrophysiology, calcium transients, metabolism *etc*). may be differentially altered by each EV type which would not be captured by the LDH assay. Some of the identified kinases such as CAMK2, PDHK and DMPK are known to participate in these processes. Another limitation of the study is that we examined a single time point which may have missed early kinase responses to the EVs. However, since the EVs remained incubated with the cells, and effectively prevented damage, for the entire hypoxia duration, the results still reflect the effects of EV-mediated protection. Thus, the absence of AKT phosphorylation at 12h, despite ongoing EV-mediated protection, suggests that cardioprotection can be achieved without sustained AKT activation. If earlier kinase activation was missed, this would have been transient, and future studies could explore additional time points and the kinetics of EV activities on cells. It should also be noted that while this study used an *in vitro* model to specifically examine human cardiomyocytes, the *in vivo* response to MI and EV therapy involves multiple cell types, including cardiac macrophages, fibroblasts, endothelial cells and circulating cells, all of which can internalize EVs ^53^. Lastly, the array used here was restricted to serine and threonine kinases, and does not include tyrosine kinases.

## Conclusion

Here we present kinase activation profiles of metabolically matured human iPSC-derived cardiomyocytes under hypoxic conditions following treatment with extracellular vesicles prepared from serum, platelet concentrates, bone marrow MSCs and cardiac stromal cells. All EVs successfully protected cardiomyocytes from prolonged hypoxic injury but each had distinct effects on intracellular kinase activities. The results showed that blood-derived EVs increased total kinase activity whereas cell-derived EVs decreased kinase activity. In particular, the AKT pathway was consistently ranked as important in EV-mediated protection, though ELISA did not find increased levels of Ser473 phosphorylation in treated cells. Further research could address the temporal dynamics of kinase activation, the role of phosphatases, and effects on other cardiac cell types.

## Materials and Methods

### Cell culture, iCM maturation and characterization.

Human induced pluripotent cell-derived cardiomyocytes (iCM), differentiated from a healthy donor (33-year-old female), were obtained from the Human Disease iPS Cells Service Consortium, Academia Sinica, Taiwan. The consortium operates under IRB approval, protocol ID AS-IRB-BM-14055 and informed consent was obtained from the donor. The donor line (ID IBMS-iPSC-001-02) is deposited with the Bioresource Collection and Research Center (BCRC), Hsinchu, Taiwan. iCMs were seeded onto Growth Factor Reduced Matrigel (Corning, 354230)-coated (1:150) plates at a density of 170,000 cells/cm^2^ based on the supplier protocol. Basal medium was RPMI 1640 medium (Thermo, 11875085), with B-27 supplement (Thermo, 17504044). Cells were kept in seeding medium (+ 10 μM Y-27632 ROCK inhibitor (Selleck Chemicals, S1049) for two days, then changed to maintenance medium (RPMI, B-27) for two additional days, following standard procedures.^54^ Thereafter, cells were switched to fatty-acid maturation medium (FA-medium) comprising RPMI, B-27, galactose (10mM), carnitine (120μM), palmitic acid (50μM), oleic acid (40μM), linoleic acid (22μM), Triiodothyronine (T3) sodium salt (0.1μM), dexamethasone (1μM), and 2% (v/v) EV-depleted FBS (Thermo, A2720801).^36,42^ The final DMSO concentration was below 0.25% (v/v). FA-Medium was changed every other day and iCMs were matured for a total of 17 days. Cardiomyocyte maturation was confirmed by RT-qPCR using primers (50 nM) shown in **Supplemental Table 1**. iCM purity was measured by flow cytometry for cardiac troponin I (Abcam, ab314676), with isotype IgG as a negative control.

### RT-qPCR.

RNA was isolated using RNeasy kits (Qiagen, 74106) then 50ng RNA was reverse transcribed using SuperScript IV (Thermo, 18-091-050). The cDNA was diluted 1:10, with 1μl used per qPCR Reaction (reaction volume of 11μl) using the SYBR Green system (Thermo, 43-687-08) on a StepOnePlus thermocycler. Cycle conditions were 10 min 95°C, 40x cycles of 15 sec 95°C and 1 min 57°C, followed by 15 sec 95°C, 1 min 57°C and then raising the temperature with a dT of + 0.5°C/sec for melting curve analysis. Reactions without cDNA were carried out and were confirmed to lack amplification for all primer sets.

### EV isolation and characterization.

EVs were isolated using the most common methods for each source; size exclusion chromatography for platelet concentrates and serum (small volumes, high protein concentration, high background protein) and ultracentrifugation for conditioned cell culture medium (large volume, low concentration). Platelet concentrate-derived EVs (PC-EVs) were isolated from outdated (5 days after collection) platelet concentrates (PCs) obtained from a blood bank, following published methodology ^55^. Ethical approval for their use in research was obtained from Taipei Medical University Institutional Review Board (TMU-IRB), protocol N202401010. In brief, pooled PCs were serum converted using calcium chloride, centrifuged, and EVs were isolated by size-exclusion chromatography (qEV 35nm Gen 2, Izon) then concentrated using centrifugal filters (Merck, UFC8050) as previously described ^10^. Serum EVs (S-EVs) were isolated from a healthy donor (female, 32 years old) following a 12h overnight fast, also using the qEV system. IRB approval (N202104120) and informed consent from the donor was obtained. Bone marrow mesenchymal stromal cells (BM-MSCs) were purchased from Lonza and grown in defined medium (Lonza, PT-3238) with growth supplements (Lonza, PT-3011). Cardiac stromal cells were isolated and cultured as previously described ^56,57^. Cardiac tissue collection was approved by TMU-IRB, protocol N201910027, and the donor provided informed consent. For preparation of conditioned medium, cells were expanded in 15 cm dishes and were 70–80% confluent, then switched into serum-free medium, with medium collected daily for three days. All cells were between passage 3 and 5. EVs were isolated from a pooled total of 300 ml conditioned medium using sequential centrifugation; (500g 10 min then 3,000g 20 min), and ultracentrifugation (100,000g 16h). The pellet was resuspended then ultracentrifuged again (100,000g 90 min) and the final pellet was resuspended in 200 μl of 0.1 μm-filtered PBS. For all EV isolates, the mean particle count and particle diameter were measured by nanoparticle tracking analysis (NTA) using a Malvern NS300 system. Samples were prepared for cryoEM by spotting the EV solution onto carbon grids and processed using VitroBot, then visualized using a Tecnai F20 microscope. These processes were carried out by a core facility technician.

### Hypoxic injury and EV treatment model.

To induce hypoxic injury, culture medium was replaced with serum-free RPMI 1640 with B27 which had been pre-acclimatized in a hypoxic incubator. Medium contained either 100 μg EV protein per ml, adjusted to equalized particle counts, or an equal volume (2% v/v) of the EV vehicle (0.1 μm-filtered PBS) as a control for the duration of hypoxia. This dose was based on previous studies ^9–11^. Hypoxia (< 1% O_2_) was maintained for 12 hours using the Anaeropack (Mitsubishi Gas Chemical) system in an airtight jar at 37°C, verified by oxygen-sensitive strips in the chamber. Experimental wells were surrounded by wells containing PBS to prevent evaporation.

### Cardiomyocyte injury and viability measurements.

iCM damage was detected by measuring LDH release into culture medium, which is a sensitive metric of membrane disruption and is used to measure cumulative damage.^37^ After the hypoxic period the supernatant was collected and LDH was measured (Dojindo, CK12-20). Blanks comprising basal medium with EVs (i.e. no cells) were also measured and the values were subtracted. The EV populations were also confirmed not to affect the LDH assay. For validation of iCM live/dead status, iCMs were stained with trypan blue.

### Protein harvesting for kinome array.

iCMs were processed following the “Protocol for Kinase Profiling of Cell Lines or Purified Cells” Version 4.1 from PamGene International B.V. Solutions, plasticware and centrifuges were pre-cooled to 4°C and all work was carried out on ice. Cells were washed gently with PBS two times to remove culture medium, lysed for 20 mins in 150 μl lysis buffer (Thermo, 78503) with (1x) protease inhibitor (Thermo, 87785) and phosphatase inhibitor (Thermo, 78420), then collected into fresh 1.5 ml Eppendorf tubes. After centrifugation (12,000g for 15 mins at 4°C) the supernatant was collected and aliquoted into fresh tubes. Protein was quantified using a Pierce BCA Protein Assay Kit (Thermo, 23227).

### Kinome array.

Kinase activity was measured using the PamStation 12 platform and STK (serine threonine kinase) PamChip^®^ peptide arrays. The chips contain short peptides which represent 144 putative phosphorylation sites in human proteins, thus acting as substrates for kinase enzymes present in the sample. An antibody against phosphorylated Ser/Thr and secondary FITC-Antibody is used to quantify the phosphorylation signals over time. Regression slopes of exposure times and incubation duration are used to calculate phosphorylation levels of each substrate. For sample loading, chips were blocked with 2% (v/v) BSA, and iCM lysate (1 μg total protein in 10μl) was added with kinase buffer with phosphatase inhibitors, 400μM ATP and FITC-conjugated antibodies, provided with the kit. The protein samples for the array were formed by pooling 4 biological replicates of each condition proportionally to their protein concentration. These were then split across multiple PamChips as technical replicates (n = 6 for hypoxia + vehicle and normoxia, n = 3 for each EV treatment group). This protocol for pooling samples was based on Pamstation 12 standard protocols, and previous studies, and is designed to control in-assay variability ^44,58^. The BioNavigator software package (PamGene) was used to convert the captured images to numerical values based on the intensity levels. The layout of the samples is shown in **Supplemental Fig. 3**.

### Upstream Kinase Analysis.

Differences in phosphorylation of reporter peptides on the STK chips were analyzed by multiple software packages: Kinome Random Sampling Analyzer (KRSA), Upstream Kinase Analysis (UKA), Post-Translational Modification-set Enrichment Analysis (PTM-SEA) and Kinase Enrichment Analysis (KEA3), as previously described. KRSA applies a random resampling approach to the list of differentially phosphorylated peptides, then assigns a score for each kinase family ^45^. The BioNavigator Upstream Kinase Analysis (UKA) tool was used to provide a scored ranking of implicated kinases, based on the specificity of the peptides mapped to the kinases and the significance of phosphorylation changes. Kinase Enrichment Analysis Version 3 (KEA3) is a web tool uses corresponding proteins of the top differentially phosphorylated reporter peptides as the input ^46^. PTM-SEA uses differentially phosphorylated sites as the input and utilizes an empirically determined collection of phosphosites to conduct an analysis akin to gene-set enrichment analysis (GSEA), producing a set of Enrichment Scores ^47^.

### Integration of upstream kinase assignments across packages.

The Creedenzymatic R package (https://github.com/CogDisResLab/creedenzymatic) was used to aggregate results from KRSA, UKA and KEA3 by combining, harmonizing and visualizing the results with percentile rank normalization.^44,58^ Kinases were mapped to the official HUGO Gene Nomenclature Committee (HGNC) symbols and subfamilies.

### Kinase activity.

Changes were assessed by calculating log2fc of phosphorylation levels for each specific substrate. Substrates with log2fc > 0.2 were assigned a value change of + 1, those between – 0.2 and 0.2 were assigned a value of 0, and those with log2fc < 0.2 were assigned a value of −1. Subsequently, the values for all substrates associated with a particular kinase were averaged. A direction of change was determined based on whether the average value was above or below zero.

### ELISAs.

AKT1/2/3 phosphorylation (Ser473) of iCM lysates was measured using a semi-quantitative ELISA-based kit (Thermo, 85-86042-11) following the manufacturer protocol. Inbuilt positive and negative controls were validated and all test samples were within range of the assay.

### Other data handling and statistics.

Statistical tests were performed in Graphpad Prism 10. Specific tests used and the samples which were compared are described in the figure legends. Figures show P values for each comparison. The heatmap of KRSA Z scores in Fig. 5 was plotted using the ComplexHeatmap R package. iCMs were metabolically matured in 2 separate batches then pooled and seeded into 96w plates for LDH assays and 24w plates for kinase assays. PC-EVs were isolated from one batch of platelet concentrate, and S-EVs, MSC-EVs and CSC-EVs were obtained from a single donor. Kinase samples were prepared by pooling together 4 separate iCM lysates then splitting the protein lysate across the wells of the kinase array (as shown in **Supplemental Fig. 3**), in line with reccommendations from Pamgene and previous publications.

## Supplementary Material

This is a list of supplementary files associated with this preprint. Click to download.

• 20260109CzosseckKinaseSupplemental.docx

## Figures and Tables

**Table 1.** Table showing mean size, particle concentration and protein concentrations of samples. The purity is shown by particles/μg of EV protein. Here, ‘sample’ refers to the final EV isolate in 200 μl, while ‘original’ is adjusted to the unprocessed sample volume.

EV type	Size ± SD (nm)	Original volume (ml)	Sample particle count (/ml)	Original particle count (/ml)	Sample protein concentration (μg protein/μl)	Purity (Particles/μg protein)
S-EV	133.1 ± 0.2	0.5	8.14E+11	3.26E+11	10.242	7.95E+10
PC-EV	112.9 ± 0.7	0.5	2.02E+12	8.08E+11	39.564	5.11E+10
CSC-EV	143.7 ± 2.9	300	1.53E+11	1.02E+09	2.056	7.44E+10
MSC-EV	162.1 ± 6.4	300	1.63E+11	1.09E+09	0.900	1.70E+11

**Table 2.** KRSA Z scores, showing 5 highest kinase families per comparison. Together, the results showed that hypoxia resulted in broad suppression of total iCM kinase activity which may be due to the relatively extended 12h hypoxia model. Among the decreased activity, PKCH, PAKB and PKN families were the most significantly affected.

Norm v. Hyp_veh		Hyp_veh vs. Hyp_S-EV		Hyp_veh vs. Hyp_PC-EV		Hyp_veh vs. Hyp_CSC-EV		Hyp_veh vs. Hyp_MSC-EV	
Kinase	Avg.Z	Kinase	Avg.Z	Kinase	Avg.Z	Kinase	Avg.Z	Kinase	Avg.Z
PKCH	2.72	FRAY	1.47	PKCH	2.42	PAKB	2.06	PAKB	2.07
PAKB	2.06	WNK	1.38	PAKB	1.79	PKCH	1.98	AUR	1.74
PKN	1.97	AKT	1.20	FRAY	1.38	AUR	1.84	PKCH	1.70
FRAY	1.82	PKN	1.15	AKT	1.23	PKN	1.59	FRAY	1.25
WNK	1.42	PAKB	0.76	PKN	1.06	VRK1	0.98	DMPK	1.16

## Data Availability

Experimental data are presented in the manuscript and the attached files. All individual data points are shown on graphs and no samples were excluded from analysis. High-throughput kinome array data and subsequent analyses are publicly available on Github: https://github.com/CogDisResLab/lundy-hypoxia-cardiac/tree/main. In addition, KRSA (https://github.com/CogDisResLab/KRSA) and Creedenzymatic (https://github.com/CogDisResLab/creedenzymatic) packages are publicly available on Github. miRNA cargo of S-EVs, PC-EVs, CSC-EVs and MSC-EVs are also available in previous publications.^9–11^
